# Recognition of Famous and Unfamiliar Faces among Patients Suffering from Amnesia Mild Cognitive Impairment (AMCI) and Alzheimer’s Disease

**Published:** 2019-07

**Authors:** Fahimeh Rahmani, Majdoddin Fathi, Maryam kazemi, Elham Bahadori

**Affiliations:** 1 Department of Psychology, Neuropsychological Assessment in Memory Clinic, University of Guilan, Rasht, Iran.; 2 Department of Psychiatry, Psychological Counseling in Rasti Clinic, Shiraz University of Medical Sciences, Shiraz, Iran.; 3 Department of Psychology, Allameh Tabataba'i University, Tehran, Iran.; 4 Neuropsychological Assessments in Mehr Clinic, Shiraz, Iran.

**Keywords:** *Alzheimer’s Disease*, *Amnesia*, *Face Recognition*, *Famous Faces*

## Abstract

**Objective:** Memory assessment for the early diagnosis of cortical dementia is a complicated process which depends on important factors such as facial recognition and naming. These factors could be considered to carry a predictive power to detect neurodegenerative disorders. The present study aimed to study and compare naming or recognizing famous faces with the recognition of newly learned faces among patients with Amnesia Mild Cognitive Impairment (AMCI) and Alzheimer’s disease.

**Method** **:** To collect data, 60 AMCI patients, 62 patients suffering from Alzheimer’s disease, and 63 cognitively healthy individuals were assessed using Wechsler Memory Scale-III Faces test (WMS-III faces) and Famous Faces test.

**Results: **The results of one-way ANOVA indicated that the patients suffering from AMCI and Alzheimer’s disease scored significantly worse than the control group on naming (p < 0.001), recognition (p < 0.001) section of the Famous Faces test, and immediate or delayed recognition on the WMS-III Faces test (p < 0.001). Also, the obtained results showed that the patients groups received lower scores on WMS-III Faces compared to the Famous Faces test.

**Conclusion: **The results of this study suggested that the unfamiliar and Famous Faces tests allow the quantification of patients’ face recognition and name recall abilities which, in turn, makes it possible to make more accurate predictions about cases of dementia. These tests can be used for clinical and research purposes to screen those who may be prone to dementia and need further neuropsychological assessment.

Every person has an ability to recognize different individual faces despite structural resemblance or major and minor differences (1). Also, of significant importance is one’s ability to recognize and remember a large number of people’s faces, as this is an essential ability in order to interact with others and perform various everyday functions (2). 

Some studies have shown that the process of remembering well-known and little-known faces are essentially different. In fact, when people try to recognize new faces, the external (e.g., hairstyle and ears) and internal (e.g., nose, eyes, and mouth) facial features are analyzed in more detail, whereas when recognizing familiar faces, the whole face is taken into account (3-4). In addition, while external and internal features play equally important roles in the recognition of unfamiliar faces, internal features are more important than external features when remembering familiar faces (4). In fact, familiar faces memory consists of three main subcomponents: one; face recognition, two; name recall, three; and the retrieval of person-related information. 

The first of these three issues, face recognition, is the cognitive process dealing with how people structurally encode the face, a multifaceted aspect of a human’s appearance subjected to the analysis of various facial features, such as the nose and eyes, in order for a person to distinguish one face from another. This analysis of structural information pertaining to the face occurs right at the beginning. 

In some rare cases, the process of integrating various facial features may be hampered as a result of difficulties in visuo-spatial perception resulting from the deterioration of the cortex. This analysis of facial features revolves around familiarity judgment, during which a given face is compared with the facial structures which an individual has previously encountered and registered in their memory. Should this analysis yield a result belonging to that of a person whom the individual is familiar with, a certain area of the brain dealing with face recognition is initially activated. This is followed by a process by which the name associated with that particular facial structure and stored in the brain is accessed and produced, which is typically known as name recall. Once the person’s name has been retrieved, other information related to that person which has previously been registered in the brain is also accessed. Such knowledge can refer to many different things, such as their relationship with the individual or their job. In other words, after successfully recognizing another person’s face, the brain attempts to retrieve the information about the person’s name as well as other semantic knowledge pertaining to that particular face (5).

Thus, when we see an individual we know personally, the process of identification is only partially based on the individual’s physical appearance; in fact, to a large extent, it depends on our knowledge of that person (e.g., personality, attitudes, way of thinking, and facts about their life), feelings, and social relationship (6). The general consensus is that the process of retrieving a person's identity information from one’s long-term memory is automatic (7). However, unfamiliar stimuli, such as unfamiliar faces or objects, are considered new information, which will need to undergo the processes of encoding, storage, or retrieval of memory, and thus can be influenced by aging (8). The findings of several studies have indicated that people are able to identify familiar faces better compared to unfamiliar faces (4, 6).

Considering the complicated processes of recognizing familiar and unfamiliar faces, it is evident that its indexes, including recognition, can carry a predictive power to detect neurodegenerative disorders (dementia) in its early stages.

Amnestic mild cognitive impairment (AMCI) is a transitional period between the normal aging and the initial stages of Alzheimer’s disease (2, 9, 10, 11), during which an individual may complain about cognitive impairments, including deficits in episodic memory (relating to new information) and semantic memory, that is, semantic knowledge retrieved from long-term memory (2, 10). The possibility that an individual with AMCI develops Alzheimer’s disease is 10%-15% each year, whereas this figure is significantly smaller among cognitively normal older adults (1%-2%). Now that researchers have identified AMCI as a stage prior to Alzheimer’s disease, the accurate differentiation of persons who are expected to suffer from mental degeneration from those who are not is now more important than ever (2, 10).

Alzheimer’s disease is an ongoing neurodegenerative disorder identified by the degeneration of both synapses and cortical neurons as well as the emergence of amyloid deposition and neurofibrillary tangles. As the condition progresses and begins to affect areas of cortical association, this gradually leads to dementia syndrome causing deficits in attentional and executive functions (e.g., planning and the execution of goal-directed plans after goal formulation), semantic memory (e.g., knowledge of faces, words, and objects), language, praxis, and constructional and visuospatial capabilities (12). Several studies have found that individuals with AMCI or Alzheimer’s disease scored significantly worse than the cognitively healthy participants on recognizing famous faces (9, 10, 13, 14, 15) and unfamiliar ones (2, 16). However, few studies have shown that impairments of facial memory can affect both episodic and semantic memory performance simultaneously in patients with AMCI or Alzheimer’s disease.

Considering what has been discussed thus far, it can be concluded that the early detection of Alzheimer’s disease is of utmost importance. As such, significant amounts of energy and time have been poured into the development of a diagnostic tool capable of detecting the initial cognitive changes characterizing Alzheimer’s disease while also being able to distinguish between various distinct phenomena, including normal aging, early Alzheimer’s disease, other brain disorders leading to loss of memory and, in particular, mimics of early-stage dementia, such as depression. However, if such a tool is to achieve widespread acceptance, it must enjoy a high degree of test-retest reliability and be relatively easy to administer, while also being cheap and simple (12). In this regard, researchers have performed a large amount of research on AMCI, particularly on the nature and predictive power of visuospatial impairments. Nevertheless, there is not much information on the impact of AMCI on episodic memory for unfamiliar faces and whether this issue could be a determining factor in diagnosing the condition and understanding whether it is the normal decline in cognition associated with aging or a case of AMCI or Alzheimer’s disease (2).

Therefore, studying and comparing the recognition of newly learned faces with recognition or naming of famous faces provides an opportunity to expand the current pool of knowledge about visual memory performance more comprehensively and examine both episodic and semantic memory performance simultaneously in patients with AMCI or Alzheimer’s disease.

Despite the aforementioned importance of the process of naming and distinguishing faces among patients with dementia (especially Alzheimer’s and early-age dementia), very few studies have been conducted on this topic. Thus, in this study, considering this gap and the need to further study the tests that are able to investigate the process of recognizing familiar and unfamiliar faces, Wechsler Memory Scale-III Faces test (WMS–III Faces for unfamiliar faces) and the Famous Faces test were used in patients with AMCI and Alzheimer’s disease.

Participants

This cross sectional study was performed on 122 patients (60 suffering from AMCI and 62 from Alzheimer’s disease) and 63 cognitively healthy participants aged 57 and 88 years without any neurodegenerative problems. All participants were assessed between January 2014 and June 2018 at 5 nursing homes affiliated to Shiraz Welfare Organization as well as geriatric, psychiatric, and neurological clinics in Shiraz, Iran. 

The controls voluntarily participated in the study and were assured that their information would remain confidential. All participants were fluent Persian speakers and had undergone at least 6 years of formal schooling. Consent forms were completed by the authorized caregivers of the patients and the members of the control groups themselves.

## Materials and Methods

***WMS-III Faces Test***

WMS-III Faces is a recognition memory test which contains 2 booklets of color photographs of different faces. In the first phase, the examiner asks the participant to see and remember 12 different new faces at a rate of 2 or 3 seconds per face. The pictures are selected with a focus on the faces, meaning that none of them have any overly distinctive features, such as heavy make-up, scars, or facial hair. Then, for the immediate recognition test, 24 faces, consisting of the original 12 in addition to 12 new faces, are presented immediately after showing the last face and asks the participants to recognize which were seen previously. Next, the participants are given advance notice that they will have 20 minutes for the delayed recognition test. Finally, the 24 faces are presented again and the participants are asked to identify those seen previously. One point is awarded for every correct response and the raw scores range from 0 to 24 for both the immediate and delayed recognition portions of the test.

In those instances, when the participant was not sure of the correct response, the examiner would ask them to make the best choice. If a participant said that he/she did not know what the answer was or appeared to be merely guessing and became unwilling to proceed with the rest of the test, the test was halted. However, in the event that the participant admitted to guessing but still wanted to continue (as may occur even with healthy elderly people), the full test was performed.

Both of the correlation coefficients between the immediate and delayed recognition scores obtained on the WMS-III Faces standard and alternative images were statistically significant (20 men and 20 women; mean age = 44.3; SD = 7.21). In fact, there were significant positive correlations between the immediate and delayed recognition scores on the standard test as well as the test consisting of alternative images (r = 0.768, p < 0.001; r = 0.591, p < 0.001, respectively). 

***Famous Faces Test***

Twenty-five images of famous faces were downloaded from the internet and used to develop the Famous Faces test. The images were selected based on the person’s popularity in the media and press at the time when the person is best-known. Forty cognitively healthy people with an average age of 41 years were presented the selected images to ascertain that the difficulty of the test was at an appropriate level. The accuracy scores for all participants in recognizing the test ranged from 85% to 100% (M = 97.4; S.D. = 2.84, respectively). None of the errors were systematic; therefore, they were not discarded and were instead used to assess the patients of the present study as well as the demographically similar control group. The selected images of famous faces seem to develop a novel method of measuring one’s ability to recognize famous faces, as they contain items which have been designed with the culture and age of those people who may be prone to dementia.

Correctly recognizing the faces was used for scoring purposes. Two points were awarded for recognition when at least 2 pieces of information about the famous person were provided, but this was reduced to 1 point if only 1 detail was given. Only details which could not be interpreted from the picture itself, such as sex, were taken into account. However, 2 full points were given for the recognition score if the participant was able to recall the person’s name, regardless of whether they could remember the famous person’s full name or only their first or second name. The participants were shown the faces one by one and asked to recognize and provide a description.

***Reliability***

The results of alternative form reliability showed that both the correlation coefficients between the naming and recognition scores obtained on the Famous Faces test (standard test and alternative images) were statistically significant (24 men and 21 women; mean age = 40.1; SD = 9.54). In fact, there were significant positive correlations between the naming and recognition scores on the standard test as well as the test consisting alternative images (r = 0.880, p < 0.001; r = 0.862, p < 0.001, respectively).

***Construct Validity***

The results of the analyses showed that the positive correlations between the total error of the Famous Faces test and the total error of immediate and delayed recognition on the WMS-III Faces test were statistically significant (r = 0.711, p < 0.001; r = 0.778, p < 0.001, respectively). 

Next, correlation coefficients for the total error of the Famous Faces test with Total Trial 1-5, Short-Delay Free Recall, Short-Delay Cued Recall, Long-Delay Free Recall, Long-Delay Cued Recall, Long-Delay Yes/No Recognition and Total Learning Slope of the SVLT were significantly negative (r = -0.457, p < 0.001; r = -0.330, p < 0.001; r = -0.304, p < 0.001; r = -0.328, p < 0.001; r = -0.420, p < 0.001; r = -0.276, p < 0.001; r = -0.294, p < 0.001, respectively). Also, there were significant positive correlations between the total error of the Famous Faces test and the false positive of the SVLT (r = 0.269, p < 0.001).

***Procedure***

All participants in the Alzheimer’s disease group were selected from the geriatric, psychiatric, and neurological clinics, all of whom had been diagnosed beforehand using MRI and clinical judgments done by a neurologist. 

Despite no a specific test to confirm an initial diagnosis of Alzheimer’s disease and AMCI, many doctors diagnose these diseases based on approaches developed by a team of international experts. For example, Jak-Bondi diagnostic techniques for AMCI are a decline in cognitive functions (between -1 and -1.5 standard deviations) on at least 2 neuropsychological tests (17). In this study, the criteria for the diagnosis of AMCI and Alzheimer’s disease were also made by 2 neuropsychological tests: Shiraz Verbal Learning test (SVLT) (18) and Mini Mental Status Exam (MMSE) (19). Those patients whose MMSE scores were between 18 and 22 were the target of the study, as they were diagnosed with mild Alzheimer’s disease or AMCI. The subscales scores of the SVLT (Total Trial 1–5, Short-Delay Free Recall, Short-Delay Cued Recall, Long-Delay Free Recall, Long-Delay Cued Recall, Long-Delay Yes/No Recognition, and Total Learning Slope) for the participants with AMCI were also identified between -1 and -1.5 standard deviations greater than the mean. However, the cutoff levels for cognitive deficits in the SVLT scales for patients suffering from Alzheimer’s disease were greater than -1.5 standard deviations above the mean. 

Furthermore, the group of healthy volunteers was selected from a nursing home affiliated to Shiraz Welfare Organization. Also, to obtain demographic information about the participants, they were asked to take part in a clinical interview, after which they were assessed using the SVLT and MMSE. Finally, their facial memory capacity was evaluated using WMS-III Faces and the Famous Faces test. All participants were assessed in a peaceful separate room involving 1 trained examiner at a time.

***Statistical Analyses***

The descriptive statistics for age, years of formal education, SVLT scales, and MMSE test were obtained for both the patients and the control groups. The WMS-III Faces immediate, delayed recognition scores, the naming and recognition portion of the Famous Faces test, and the subscales scores of the SVLT in 3 groups were then compared using one-way ANOVA. Furthermore, a paired t test was used to compare 3 pairs of scores (pair 1: immediate and delayed recognition of WMS-III Faces; pair 2: immediate recognition of WMS-III Faces and the recognition portion of the Famous Faces Test; and pair 3: delayed recognition of WMS-III Faces and the recognition portion of the Famous Faces test) in patients with mild Alzheimer’s disease or AMCI. Pearson’s correlation analysis was also used to analyze the correlation coefficients of the scores obtained on the total error of the Famous Faces test, total error of the WMS-III Faces test, and the subscales scores of the SVLT. All statistical analyses were performed using SPSS 16.0 software. Significance level was set at 95% for both WMS-III Faces and the Famous Faces test.

## Results

***Descriptive statistics***

The demographic characteristics of the participants are shown in Table 1. There were no significant differences in age and the years of formal education between the 3 groups [F (2, 2.218), p = 0.11; F (2, 2.709), p = 0.07; respectively]. The results of one-way ANOVA indicated that differences in MMSE mean scores between the 3 groups were significant [F (2, 166.435, p < 0.001]. A Tukey HSD (Honestly Significant Difference) was also used to indicate which groups were significantly different from one another. Also, the results of the post hoc test for the MMSE test showed a significant interaction among all pair of groups (Tukey's HSD, p < 0.001).

Table 2 demonstrates the mean raw scores of the female and male participants on the subscales of the SVLT according to 3 groups. The results of one-way ANOVA revealed that nonclinical participants and those with Alzheimer’s disease and AMCI had a significant impact on the Total Trail 1-5 or immediate memory, Short-Delay Free Recall, Short-Delay Cued Recall, Long-Delay Free Recall, Long-Delay Cued Recall, Long-Delay Yes/No Recognition, and False Positive and Total Learning Slope [F (2, 177.809), p < 0.001; F (2, 152.957), p < 0.001; F (2, 206.205), p < 0.001; F (2, 196.403), p < 0.001; F (2, 218.038), p < 0.001; F (2, 38.804), p < 0.001; F (2, 52.151), p < 0.001; and F (2, 76.955), p < 0.001; respectively]. The results of Tukey's HSD were also significant in all pair of groups (Tukey's HSD, p < 0.001).

***Famous Faces Test***

The cognitively healthy participants gained the average error total scores of 0.14 and 1.49 in both the naming and recognition segments of the Famous Faces test (Table 1). The results of one-way ANOVA indicated that the naming and recognition scores of the AMCI and Alzheimer’s patients were significantly lower compared to the normal elderly participants [F (2, 88.989), p < 0.001; and F (2, 59.449), p < 0.001; respectively]. The results of Tukey's HSD were also significant in all pair of groups (Tukey's HSD, p < 0.001). 

***WMS-III Faces***

The error mean scores of the WMS-III faces test achieved by AMCI, Alzheimer’s patients, and control group are presented in Table 1. The results of one-way ANOVA also indicated that the immediate and delayed recognition error scores on WMS-III Faces for 2 patient’s groups were significantly greater compared to the elderly participants [F (2, 91.9), p < 0.001; and F (2, 115.575), p < 0.001; respectively]. The results of post hoc test for the immediate and delayed recognition error scores on WMS-III Faces showed a significant interaction in all pair of groups (Tukey's HSD, p < 0.001). 

***WMS-III Faces Immediate/Delayed Recognition Comparison Study***

The results obtained by the paired t test indicated that the participants with AMCI and Alzheimer’s disease achieved significantly higher error mean scores in the delayed recognition portion of WMS-III Faces compared to the immediate recognition section of this test (t = -8.78; p = p < 0.001 and t = -4.73; p = p < 0.001, respectively) (Tables 3 & 4).

***Famous Faces Test/WMS-III Faces Recognition Comparison Study***

The results obtained by the paired t test indicate that the patients suffering from AMCI and Alzheimer’s disease scored significantly worse on both the immediate and delayed recognition segments of WMS-III Faces compared to the recognition part of the Famous Faces test (Tables 3 & 4).

## Discussion

In the present study, a new screening test, the Famous Face test, was specifically designed for Persian-speaking population to detect face identification deficits among a group of patients with dementia. The obtained results indicated that this updated test is similar to WMS-III faces test and has the potential to become an appropriate neuropsychological instrument for the detection of face recognition impairments in patients with neurodegenerative dementias (Alzheimer’s and early-age dementia). In fact, the Famous Faces test is highly sensitive to early cognitive changes relevant to facial recognition and naming impairments which usually occur among patients with cortical atrophy, such as Alzheimer’s disease, and could pave the way for the discovery of preventive methods for these types of disorders.

The results of group comparisons revealed that the patients with AMCI and Alzheimer’s disease obtained significantly lower scores than those in the control group on both the naming and recognition sections of the Famous Faces test. These findings are in agreement with those of previous studies that have clearly indicated that the ability to recognize faces and recall names decreases noticeably as a result of neurodegenerative disorders (5, 6, 20-24). 

Remembering proper names is one of the most common complaints of the elderly which could get worse as time passes. Although this issue often manifests itself in social gatherings when an elderly person struggles to recall the name of a person he or she has just been introduced to, it is also seen when trying to recognize and name familiar faces (25). These complaints can sometimes be attributed to forgetfulness which everyone might experience in their life and is different from the memory deficits resulting from dementia. Cognitively healthy middle-aged adults may not remember their neighbor’s name momentarily, but they still know exactly who they are talking to, whereas a patient with dementia will have trouble remembering his/her neighbor’s name as well as who the person is (5).

Face-name recognition is a valid test for memory which can be challenging for people suffering from AMCI or Alzheimer’s disease (22). In fact, many studies have clearly indicated performance impairments detected by the faces and names tests can reflect changes in semantic memory (9, 10-16). Few studies have shown that impairments of face-name memory can affect both episodic and semantic memory performance. However, recognizing faces and recalling names are highly complicated cognitive processes, as there is a high degree of complexity in the similarities and differences among faces and there is an arbitrary association between faces and names. Furthermore, improving face-name memory is possible through cognitive interventions targeted to promote procedural memory, which is often preserved until the late stages of dementia (5).

The results of group comparisons revealed that the patients with AMCI or Alzheimer’s disease obtained significantly lower scores than the participants in the control group on the recognition section of the WMS–III Faces test. These findings are in agreement with those of previous studies which have clearly indicated that the ability to recognize unfamiliar faces decreases noticeably as a result of neurodegenerative disorders (2, 16, 26). This turn of events could be due to a number of factors.

A number of studies have found that in various areas of cognition, including verbal and visuospatial skills, executive function, attention and speed of perception, the presence of preclinical cognitive deficits in AMCI are evident (27). However, the most common type of memory which is damaged is episodic memory, which refers to one’s ability to recall experiences (28). One of the characteristic effects of Alzheimer’s disease as well as AMCI is the deterioration of this memory (29). Such deficits often signal the initial stages of the disease and occur as a result of significant difficulty in the encoding of new input (30).

In addition, weak performances on memory tests are often thought to be related to issues with the subcomponents of the medial temporal lobe (MTL), one of which is the hippocampus, whose primary function is the strengthening of episodic memory. According to recent neuroimaging studies, the bilateral anterior temporal lobe is activated during the retrieval of pre-existing semantic and biographical information related to famous faces (7, 24, 31, 32), whereas unfamiliar faces are recognized through the perirhinal cortex of the MTL (2). Barense et al (2012), on the other hand, has put forth the idea that tests such as WMS-III Faces can cause perceptual processing overload resulting from the presentation of numerous stimuli, and this can lead to weaker representations in the perirhinal cortex of the MTL which, in turn, impacts memory (2).

It was also found that patients with AMCI and Alzheimer’s disease perform significantly better on the recognition portion of the Famous Faces test compared to both the immediate and delayed recognition sections of WMS-III Faces. This result is consistent with previous studies (1, 3).

The fact that people in general have greater difficulty in recalling unfamiliar faces than familiar ones can be attributed to the underlying nature of the visual face memory. One’s knowledge of famous faces has been formed and consolidated over time and over the course of several encounters, thus making this information easier to access and retrieve from their visual memory when the same face is seen again. This is in contrast to the information available on relatively unfamiliar faces, as it is typically based on a rather small number of previous meetings. This may also lead to discrepancies in the processing of such information, that is, information pertaining to familiar faces is typically processed instantly irrespective of the activity at hand; however, information on unfamiliar faces needed in the recognition process is usually only accessed when it carries some sort of significance for the current task (33). 

In general, the capacity for perceiving and maintaining constant facial information which is accessed when identifying a face is an essential factor. Although unfamiliar faces are stored as images similar to the profile pictures people keep in social networking sites, a lot of facial codes along with semantic and visually-derived structural codes are activated when recognizing famous faces (1).

In conclusion, as has been pointed out by numerous studies, it should be reiterated that the elderly suffering from cortical dementia experience a significant fall in their ability to recall the names or recognize the faces of famous people and recognize unfamiliar faces. Thus, an effort should be made to develop methods capable of screening or predicting dementia while it is still in its early stages so that measures can be taken to prevent the development of this disorder and ensure that the elderly are able to enjoy normal lives longer than what has been possible thus far. Because the Famous Faces test and the WMS–III Faces test can provide quantified data on one’s ability to recall names and recognize faces according to detailed scoring criteria, they have the potential to be used as a clinical instrument to predict dementia in Persian-speaking populations.

Nevertheless, as is the case with any study, there were several limitations which make it necessary to conduct further research. In particular, it is beneficial to replicate the study with a larger sample size. Moreover, comparison of facial recognition and name recall among patients suffering from different types of cortical and subcortical dementia is of high valuable.

**Table1:** Demographic Characteristics of the Amnestic Mild Cognitive Impairment (AMCI), Alzheimer’s Disease, and Healthy Older Groups

**Variable**	**Healthy Older ** **Adults n = 63**	**AMCI n = ** **60**	**Alzheimer’s ** **Patients n = 62**	**F**	**Sig**
age	70.5 (7.56)	71.5 (7.07)	73.4 (8.52)	2.218	0.11
Years of Education	11.9 (3.18)	11.50 (3.76)	10.4 (3.84)	2.709	0.07
MMSE	26.9 (2.37)	21.3 (2.89)	18.3 (2.80)	166.435	0.001
The total error of immediate recognition in the WMS-III faces	4.10 (2.03)	8.10 (2.57)	9.90 (2.82)	91.9	0.001
The total error of delayed recognition in the WMS-III faces	5.06 (2.28)	9.70 (2.35)	11.1 (2.35)	115.575	0.001
The total error of Famous Faces Naming	1.49 (1.40)	4.56 (2.33)	6.30 (2.20)	88.989	0.001
The total error of Famous Faces Recognition	0.14 (0.47)	2.10 (1.96)	3.43 (2.17)	59.449	0.001

Notes: Data are presented as mean (standard deviation).

MMSE: Mini Mental Status Examinations

**Table2:** Mean and Standard Deviation of the SVLT for the AMCI, Alzheimer’s Disease, and Healthy Older Groups

**SVLT variable**	**Gender**	**Healthy older ** **adults n = 63**	**AMCI** **n = 60**	**Alzheimer’s ** **patients n = 62**	**F**	**Sig**
Total Trails 1-5	men	45.1 (7.92)	33.9 (8.50)	22.8 (8.25)	177.809	0.001
	women	53.4 (7.37)	39.1 (6.36)	23.7 (5.21)		
Short-Delay Free Recall	men	8.65 (3.17)	4.42 (2.10)	2.40 (1.97)	152.957	0.001
	women	11.4 (2.77)	6.37 (2.91)	2.70 (1.13)		
Short-Delay Cued Recall	men	10.6 (2.63)	6.33 (2.23)	4.13 (1.81)	206.205	0.001
	women	12.6 (1.78)	7.51 (1.94)	4.35 (1.88)		
Long-Delay Free Recall	men	9.65 (3.03)	4.34 (3.17)	1.62 (1.99)	196.403	0.001
	women	12.2 (2.20)	7.10 (2.37)	1.70 (2.14)		
Long-Delay Cued Recall	men	10.9 (2.61)	5.85 (2.69)	3.21 (2.01)	218.038	0.001
	women	13.1 (1.75)	7.88 (2.02)	4.03 (1.80)		
Long-Delay Yes/No Recognition	men	14.2 (1.36)	11.7 (2.76)	10.9 (3.23)	38.804	0.001
	women	15.2 (1.01)	12.5 (2.70)	10.7 (3.45)		
False positive	men	0.96 (1.28)	4.52 (3.05)	8.18 (8.43)	52.151	0.001
	women	0.45 (1.02)	4.22 (3.75)	12.3 (7.10)		
Total Learning Slope	men	1.47 (0.46)	0.85 (0.61)	0.48 (0.34)	76.955	0.001
	women	1.72 (0.54)	1.27 (0.44)	0.49 (0.47)		

Notes: Data are presented as mean (standard deviation)

SVLT: Shiraz Verbal Learning Test

**Table3:** The Result of Paired T-Test to Compare the Error Total of the WMS-III Faces with the Famous Faces Recognition of the AMCI Group

	**Variable**	**Mean**	**Standard ** **deviation**	**Mean difference**	**df**	**T**	**Sig.**
Pair 1	Immediate recognition	8.01	2.57	-1.70	59	-8.78	< 0.001
	Delayed recognition	9.70	2.35				
Pair 2	Immediate recognition	8.01	2.57	5.90	59	23.2	< 0.001
	Famous Faces recognition	2.10	1.96				
Pair 3	Delayed recognition	9.70	2.35	7.60	59	35.7	< 0.001
	Famous Faces recognition	2.10	1.96				

WMS-III: Wechsler Memory Scale-III

AMCI: Amnestic Mild Cognitive Impairment

**Table4:** The Result of Paired T-Test to Compare the Error Total of the WMS-III Faces with the Famous Faces Recognition of Alzheimer’s Patients

	**Variable**	**Mean**	**Standard deviation**	**Mean difference**	**df**	**T**	**Sig.**
Pair 1	Immediate recognition	9.90	2.82	-1.18	63	-4.73	< 0.001
	Delayed recognition	11.1	2.35				
Pair 2	Immediate recognition	9.90	2.82	6.45	63	22.1	< 0.001
	Famous Faces recognition	3.43	2.17				
Pair 3	Delayed recognition	11.1	2.35	7.65	63	31.5	< 0.001
	Famous Faces recognition	3.45	2.17				

WMS-III: Wechsler Memory Scale-III

## Limitation

Further research is needed a larger sample of AMCI and Alzheimer’s disease patients so as to facilitate the comparison of these two groups’ performances on the recognition of famous and unfamiliar faces.

## Conclusion

Considering the obtained results, it appears that the Familiar and unfamiliar faces tests can be utilized for differentiating AMCI or Alzheimer’s disease from normal aging and it has the potential to identify risk for cognitive decline.
